# Middle Cerebral Artery Calcification: Association With Ischemic Stroke: Erratum

**DOI:** 10.1097/01.md.0000479907.99874.93

**Published:** 2016-01-22

In the article “Middle Cerebral Artery Calcification: Association With Ischemic Stroke”,^1^ which appeared in Volume 94, Issue 50 of *Medicine*, funding information was originally omitted. The article has since been corrected online.
